# Communicable disease control in China: From Mao to now

**Published:** 2011-12

**Authors:** David Hipgrave

**Affiliations:** Nossal Institute for Global Health, University of Melbourne, Melbourne, Australia; * Formerly UNICEF China Chief of Health, Nutrition, and Water and Environmental Sanitation

## Abstract

China’s progress on communicable disease control (CDC) in the 30 years after establishment of the People’s Republic in 1949 is widely regarded as remarkable. Life expectancy soared by around 30 years, infant mortality plummeted and smallpox, sexually transmitted diseases and many other infections were either eliminated or decreased massively in incidence, largely as a result of CDC. By the mid-1970s, China was already undergoing the epidemiologic transition, years ahead of other nations of similar economic status. These early successes can be attributed to population mobilization, mass campaigns and a focus on sanitation, hygiene, clean water and clean delivery, and occurred despite political instability and slow economic progress. The 10-year Cultural Revolution from 1966 brought many hardships, but also clinical care and continuing public health programs to the masses through community-funded medical schemes and the establishment of community-based health workers. These people-focused approaches broke down with China’s market reforms from 1980. Village doctors turned to private practice as community funding ceased, and the attention paid to rural public health declined. CDC relied on vertical programs, some of them successful (such as elimination of lymphatic filariasis and child immunisation), but others (such as control of schistosomiasis and tuberculosis) demonstrating only intermittent progress due to failed strategies or reliance on support by the poorest governments and health workers, who could not or would not collaborate. In addition, China’s laissez-faire approach to public health placed it at great risk, as evidenced by the outbreak in 2003 of the Severe Acute Respiratory Syndrome. Since then, major changes to disease reporting, the priority given to CDC including through major new domestic resources and reform of China’s health system offer encouragement for CDC. While decentralized funding and varying quality diagnosis, reporting and treatment of infectious diseases remain major challenges, national priority on CDC in China is high.

There are two things about modern China with which most readers will be familiar. The first is that it is the world’s most populous nation: recently released census data revealed that China’s population in 2010 approached 1.34 billion. This is below the figure of 1.4 billion anticipated, as the growth rate of 0.57% per annum has fallen substantially. China’s population, along with that in the rest of the world, began to grow very rapidly from the mid-18th century, from an estimated 177 million in 1750 to approximately 430 million in 1850 and 580 million by 1950 (1). The low annual growth rate of only 0.3% during the century to 1950 changed with the relative political stability since 1949; the population sky-rocketed in the 1950s and 1960s. This resulted in public advocacy on family planning (“later, longer, fewer”) and finally the one-child policy that has applied to around two-thirds of couples since the late 1970s (2). The need for population control in China was based not only on the formerly high fecundity of Chinese women, but also the rapid fall in the crude death rate that accompanied the establishment of the People’s Republic of China (PRC). This fall was largely due to communicable disease control (CDC).

The second familiar aspect is China’s meteoric economic development, with an average annual growth rate of around 10% over the last 30 years. China’s economic performance is now a major influence on global financial markets, with the developed world now heavily dependent on China’s continued growth. Less familiar is the fact that this stellar economic performance only commenced in the second half of the 62 years since 1949.

Both of these familiar aspects of China almost certainly depend heavily on the fact that China’s population, for the most part, became relatively healthy compared to residents in nations at a similar stage of development during the first 30 years of the PRC, and certainly much healthier that it was in 1949. By 1980, life expectancy in low-income China (67 years) exceeded that of most nations of similar gross domestic product per capita by seven years (as estimated in 1984), and indeed exceeded that of many middle-income nations (3). Although with some exceptions the health of China’s population depends now largely on control of non-communicable diseases (NCDs), the foundation of China’s population health, particularly the amazing growth in life expectancy from an estimated 32 years in 1949, depended almost entirely on CDC.

This paper provides an overview of CDC in China since the defeat of China’s Nationalists by Mao Zedong’s Communists. With regular reference to the contemporary political and economic context, it first describes what is known about disease epidemiology and causes of death before 1949, the strategies used in CDC and the major achievements made in the next 30 years. It follows with a description of the decline of CDC and community-funded public health in the context of China’s economic reform, the vertical and vertically-funded disease-control programs and, through SARS, the awakening in China of the risk posed to the people and the nation of ignoring disease surveillance and a population-level approach to public health. The paper finishes with an overview of the status of certain communicable diseases and CDC in China in 2011, and analysis of the impact of China’s current health system reforms on this issue.

## 1949 – 1979: Communicable disease control and mortality reduction on a mass scale

When the Communists founded the PRC in early October, 1949, they established control of one of the most impoverished nations on earth. After a century of domination by Europeans, the fall of the Qing Empire was followed by partial Japanese occupation and a 38-year civil war. The vast majority of the population were engaged in subsistence agriculture, and a survey on the causes of death conducted in 1929-31 revealed that more than half of all deaths were caused by infectious diseases. A list of leading health problems before 1949 (**Table 1**) is noteworthy for the virtual absence of non-communicable diseases (King and Locke, 1983 as cited in ref. 1), and rural health care was in very poor supply (4-6).

**Table 1:** Major health problems in China before 1949*

INFECTIONS			OTHER CONDITIONS
Amoebic dysentery	Japanese B encephalitis	Schistosomiasis	Bronchitis
Ancylostomiasis (hookworm)	Leishmaniasis	Smallpox	Diabetes
Anthrax	Leprosy	Syphilis	Encephalomyelitis
Ascariasis (roundworm)	Leptospirosis	Taeniasis	Fluorosis
Bacillary dysentery	Malaria	Tahyna fever/encephalitis	Kashin-Beck disease
Brucellosis	Measles	Tapeworm	Glaucoma
Cholera	Mumps	Tetanus	Goiter
Clonorchiasis (liver fluke)	Paragonimiasis	Tick-borne relapsing fever	Keshan disease
Dengue fever	Pertussis	Trachoma	Malnutrition
Diphtheria	Plague	Tuberculosis	Nephritis
Enterobiasis (pinworm)	Pneumonia	Typhoid/paratyphoid	Opium addiction
Epidemic meningitis	Polio	Typhus	Rickets
Fasciolopsiasis	Rabies	Varicella	
Filariasis	Rheumatic fever	Viral haemorrhagic fever	
Gonorrhoea	Ringworm	Viral hepatitis	
Influenza	Scarlet fever		

*Data adapted from Banister, 1987 (1).

### Early disease-control programs

The political turmoil and slow socioeconomic development in China between 1949 and 1978 obscure its impressive progress in population health during those years. The Communists were quick to make good on promises of land-reform and establishment of a national “people’s” government. In 1950 a Marriage Law was enacted, providing equal rights for women, and the first National Health Congress established a focus on rural health, disease prevention through campaigns, and collaboration between western and traditional Chinese medicine. The focus on improving rural health and on CDC persisted until the 1980s.

Early efforts in public health included work on vaccination, environmental sanitation and hygiene (including the early introduction of composting of night-soil to reduce the concentration of intestinal parasites) and the development of organized CDC programs. Incredibly, between 1950 and 1952, over 512 million of China’s ~ 600 million people were vaccinated against smallpox, massively reducing case numbers; the last outbreak of smallpox in China occurred in 1960, 20 years before global eradication (7). By 1957, more than two-thirds of China’s then ~ 2050 counties had an epidemic prevention station (EPS) or more specialized centres for the control of specific diseases (such as malaria, plague, schistosomiasis, leishmaniasis and brucellosis) modelled on those established in the Soviet Union earlier in the 20th century. Their efforts included “patriotic health campaigns” focusing on ensuring a clean environment and safe drinking water, vector control, latrine construction and human waste disposal. Each of these short-term interventions (on average twice a year, lasting for around a week) required the mass mobilization of peasants, and so served to increase the “health literacy” of the rural population (1,6-8).

Apart from targeted vaccination, other nascent disease control programs emerged. As a result, cases of typhus dropped by 95% in the 1950s, and there were also major attempts to control gonorrhoea and syphilis (considered by the communists to be social diseases associated with liberal western attitudes and affecting up to 50% of some population groups), first with imported and then domestically produced penicillin. Prostitution was also outlawed and the status of women elevated (6,9-11). Vaccination and campaigns against diphtheria and tuberculosis (TB) also commenced in the 1950s. In the late 1950s, another campaign to “exterminate the four pests” (sparrows, rats, flies and mosquitoes) was avidly implemented, albeit with major negative results when the exploding locust population decimated crop harvests, contributing to famine from 1958-1960 (1,7).

**Figure 1:**
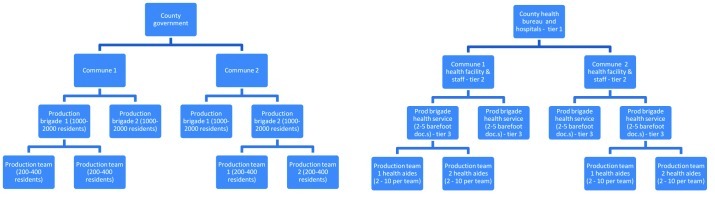
The rural government and health system in 1960s–1970s China, depicting the three-tier network.

Newborn and puerperal infection rates also decreased tremendously during this period, with the re-training of up to 750 000 traditional midwives and establishment of 2380 maternal and child health (MCH) centres by 1952. No other type of medical facility increased at this rate, and a major result was the decline in neonatal tetanus, down from up to 5% of all newborns to a fraction of this Figure (1,6.

Whilst tremendously successful, these mostly preventive care efforts, however, do not infer that rural Chinese had access to clinical care in the 1950s. Patriotic health campaigns were highly effective in CDC but were rarely sustained for more than a month; diseases not addressed by the campaigns were simply neglected and curative care was virtually unavailable outside the cities. Medical schools primarily trained doctors for hospital work. Rural Chinese basically only had access to Chinese herbal medicine and other traditional healers until well into the 1960s (1,6).

In addition, the patriotic health campaigns occurred in the context of major political instability in China. After liberation of the masses in 1949 and a period of relative self-control by peasants of their newly acquired land and produce, Mao introduced a set of disastrous social and economic policies involving community and agricultural collectivization. Motivated by jealousy of the Soviet Union and the west and his perspectives that the rural masses should be both self-sufficient and the source of grain for the cities, Mao promoted the Great Leap Forward from 1958-1960. This included new cultivation methods that failed dismally, further reducing the harvest. Impacted also by adverse weather and the locusts, the resulting famine resulted in the death by starvation of tens of millions, temporarily halting the rapid population growth wrought by successes in CDC.

### Village doctors bring curative care, knowledge and a public health approach to the masses

After the disastrous Great Leap period, Mao retreated into the political background and China entered a period of relative political quiet in the early 1960s. Collectivization was relaxed and the patriotic health campaigns continued. EPSs grew in number, reaching around 2500 by 1965 (7), and vertical CDC programs expanded. With a return to food security (albeit with rationing), population growth resumed and life expectancy continued to grow (1). However, unhappy with his perception that the revolution was faltering, development was slowing and that his own political star was fading, in 1966 Mao launched the Cultural Revolution, throwing China into a ten-year period of political and economic chaos. The Revolution was characterized by mass mobilization of urban youth against authority, closure of higher education institutions and a “return to the countryside” policy to pursue revolution as an abstract concept (6).

One positive element of this period, however, was the establishment of a village level cooperative medical scheme (CMS) managed by “barefoot doctors”, a new cadre of community-level health worker who brought basic curative care, health education and a continuous rather than campaign-style public health approach to rural peasants (12). Later hailed as the foundation of primary health care (13), China’s barefoot doctors rose in number from around one million in 1970 to a peak of around 1.8 million in 1977. Many barefoot doctors were selected from, functioned in the context of and were largely funded by local production brigades (roughly 1000-2000 people in a geographic area) or teams (200-400 people). These brigades had replaced the failed, larger communes established during the Great Leap years, and apart from their commitment to providing grain to the national coffers at fixed prices, were semi-autonomous. Other barefoot doctors were selected from among the urban youths who were “sent down” to the countryside, ill-equipped to farm but educated and literate enough to be trained in basic health care. As a result, and also because each brigade had variable financial capacity to fund its CMS, the quality of health care provided by the barefoot doctors (and an even more basic cadre of community health worker, the health aide, whose numbers added an additional 3.7 million to the community health workforce in 1970) varied widely (**Figure 1**). It also depended on the level and quality of training (which varied from one to six months in duration) and supervision. Some villages also benefited from physicians who had been sent down from the cities for ideological re-education but continued to provide health care, and also from oversight by the EPS team at county level (6,12,14).

The roles of the barefoot doctors and health aides included environmental sanitation, health education, disease screening, surveillance and control, basic clinical care or referral and family planning. CDC continued to benefit from management of water sources and disposal of human excreta (including through composting), improvements in wells, toilets, stables, cooking areas and the local environment, and specific disease control programs through reducing stagnant water, spraying and other measures to control flies, fleas and mosquitoes. Although the barefoot doctors continued the “prevention first” approach to CDC established in the 1950s under the guidance of the Patriotic Health Campaign Coordination Office (a quasi-Ministerial agency only absorbed into the Ministry of Health in 1989), clinical links were established via a three-tier referral network from village through commune to county levels, with supervision in the reverse direction. This three-tier network persists today (7,15,16).

Although politically inseparable from the prevailing harsh limitations on personal expression and movement (6), CDC in China in the late 1960s and throughout the 1970s thus benefited from a large cohort of community-level staff (health aides, barefoot doctors, sent-down physicians and also midwives) with a basic knowledge of health and hygiene (14). These cadres continued the “serve the people” philosophy of the patriotic campaigns initiated in the 1950s, but with a bottom-up rather than top-down approach (4) and, along with other determinants, especially education, contributed in a highly cost-effective way to the continually plummeting crude death and child mortality rates, rising life expectancy and to CDC in rural China.

### Perspectives on the origin of China’s village doctors

**Figure 2:**
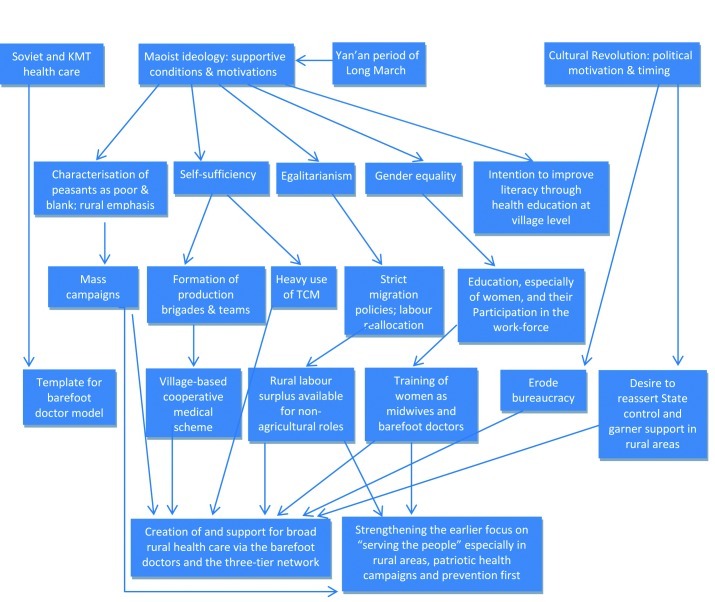
Origins and determinants of China’s barefoot doctors program (after Bien, 2008; ref 6).

The rationale for the introduction of the barefoot doctors, and their impact, has interested recent scholars, and the different perspectives are summarized in **Figure 2**. One thesis holds that they were part of Mao’s goal of improving the level of literacy in China, itself the antithesis of the contemporary philosophy that education was bourgeois (17). In support of this theory is the observation that improvements in education complemented the public health campaigns in reducing mortality (8). Another points to three influences: (i) models provided by Guomindang experiments on basic primary health care in the 1930s and 1940s, and the Soviet ‘feldshers’ (field doctors who provided primary health care at village level, supervised by trained staff at higher levels); (ii) the ideology of self-sufficiency, gender equality and egalitarianism (with the peasants as the agents, not just the beneficiaries of revolution), taken up by the Mao and the Communists in Yan’an in the 1940s (also giving rise to the preference for the traditional Chinese medicine practiced by barefoot doctors) and (iii) the political situation in the mid-1960s, which gave rise to Mao’s contention that the urban elite (including the Ministry of Health) was ignoring the backbone of the Revolution, the rural peasantry (18), and undermining his reliance on them for his own status. Having failed at commune level during the Great Leap years, self-sufficiency was instead introduced at the more stable village or brigade level, represented in the health sector by the barefoot doctors and the CMS. Whilst benefiting the health status of the population, the benefit for the nation as a whole through collectivization at this lower level was the resulting reliable supply of grain for the cities (6).

Another feature of this period that facilitated the success of the barefoot doctor movement was the surfeit of labour generated by the burgeoning population, movement restrictions that kept the rural population above 80% of the total until 1979 and the relocation of educated urban dwellers to the countryside. Sent-down physicians and urban-educated barefoot doctors made the most of the relative physical ease and prestige of their work, and the fact that income was somewhat less dependent on state-controlled grain prices (6,14).

Finally, the focus on gender equity was another significant influence on the success of the barefoot doctor movement. Although only one third of officially designated barefoot doctors were female, women made up the majority of midwives and health aides, who also functioned as barefoot doctors and contributed to CDC. Ideologies promoting female participation in the rural labour force provided the barefoot doctors program with a significant source of labour, also contributing to effective MCH programs (6).

Along with various social determinants, particularly education and the emancipation of women, the outcome of the PRC’s efforts in CDC and community-funded public health during its first 30 years are remarkable indeed, considering its relatively poor economic progress. A 1984 World Bank report suggests China was already entering the epidemiologic transition in the mid-1970s, with deaths due to communicable disease down to only 25%, compared to 44% in other low income countries and virtually all deaths before 1949 (3). Other reports document an increase in life expectancy from 35 to 68 years, a fall in the crude mortality rate of around 66% and infant mortality from around 250 to 40 deaths per 1000 live births and a decrease in malaria prevalence from 5.5 to 0.3% of the population, between 1949 and 1981 (7,14).

## Marketisation and the breakdown of community-funded primary health care in the 1980s

The introduction of market reforms in 1980 heralded the collapse of China’s brigade system, the CMS and the funding for the barefoot doctors (19), many of whom abandoned this work in favour of farming (which became more profitable with the abandonment of collective agriculture), or moved to the cities in the context of relaxed movement control (20). From 1979 to 1984, CMS coverage fell from 80%-90% of peasants to 40%-45%, and those schemes remaining offered variable and limited coverage (14). By 1986, rural CMS coverage had fallen to 9.5% (15). The number of the newly-named “village doctors” fell to around 1.2 million by 1984, and their supervision and regular retraining also decreased dramatically (14,21), resulting in falling standards despite them handling almost 50% of the nation’s clinical work. Having lost their income from the CMS, village doctors have ever since relied on generation of income from fees and the sale of drugs, resulting in abandonment of public health work and major problems with over-prescribing of drugs and inappropriate use of parenteral preparations (20-25), problems that are only now being addressed (26). Payment for health care became the responsibility of the individual; government spending on health as recently as 2008 averaged less than 1% of the national budget (27) and the plummeting affordability of health care resulted in persistently low rates of rural hospital bed occupancy (15,28) and slower declines in infant mortality and the crude death rate (7,29,30). Urban-rural disparities in health funding, facility quality, staff allocations and service uptake rose dramatically, demonstrating burgeoning inequity in China’s health sector (15,21,29). Financial decentralization was applied in both the commercial and public sectors, leaving province and county governments to mostly fend for themselves, with minimal support from the national government 814) (14); government funding as a proportion of total health expenditure fell from almost 40% in the early 1980s to below 20% by 1993 and remained below this figure until 2007 (21,31). It has risen sharply in recent years. Another source has the government’s share of total health expenditure falling from 32% to 16% from 1978 to 2002 (32).

Public health in general and CDC in particular suffered badly in this new marketised milieu, as funding for preventive health services declined and the government adopted a laissez-faire attitude to preventive health (19,33). While overall government health resources increased at an annual rate of 6% from 1980 to 1995, the rate of increase for public health services was only 4.8%. The public health share of the health budget declined from 15-18% in the 1970s to 10.6% by 1995. Hospitals were the winners, as the focus on prevention switched to treatment (19). While the falls in county level public health funding were bad, they were worse at commune (now called township) level, with funds covering less than 60% of salaries and nothing else by 1993 (34). Funding of preventive health activities at village level that characterized the barefoot doctor period totally disappeared over the 1980s, and is only now beginning to recover with China’s current health system reforms. One reason for this numeric increase but relative decline in public health funding was the increasing number of public health staff and facilities. As with curative services, government successfully reduced the cost but maintained the operation of public health services and CDC by encouraging self-sufficiency through the charging of fees for inspections and vertical programs, and there is good evidence of reduced wastage and improved productivity and efficiency in this regard (34). However, again there were problems with over-servicing of facilities who could afford the fees and ignoring weaker ones with greater problems. In food safety, this was shown by the rising incidence of hepatitis, typhoid and paratyphoid from 1979 to 1988 (19). Public preventive health activities (public goods without direct benefit to consumers) that were not profitable were often neglected or ignored; fees were even charged for vertical disease control programs (such as those against TB and schistosomiasis) despite national targets indicating their priority in the 7^th^ and 8^th^ five-year plans (7), an acknowledgement of the reliance on their implementation by staff whose participation could only be guaranteed with a financial incentive (or who charged fees regardless of services being notionally free). New charges for specific activities such as vaccination, control of schistosomiasis, TB, leprosy and also MCH reduced their uptake and impact. However, rather than cancel vaccination fees, the government introduced an immunization insurance scheme to counter falling coverage (apparently with good effect) (15), and fees for routine vaccination were only officially banned in 2007; the sale of optional vaccines (including several of the new vaccines recommended by WHO) remains a significant source of income for CDCs in China. Decentralisation of social service funding resulted in differential services according to counties’ and townships’ ability to fund them and the level of prioritization of public health by local authorities. Vertical lines of communication and control of the health system by health authorities also weakened (19). Administration of township health services gradually devolved from county to township governments, and the township health facilities divided into clinical and preventive sections, with separate funding, revenue and reporting streams (15). Most EPSs reported to local government rather than to higher levels within the health hierarchy, exacerbating the politicization of data and probability of its desensitization. Local government was usually more concerned with economic than social indicators, and disinclined to report bad news like disease outbreaks. They were also disinclined to spend public money on CDC when they could use it to make the county rich.

**Figure 3:**
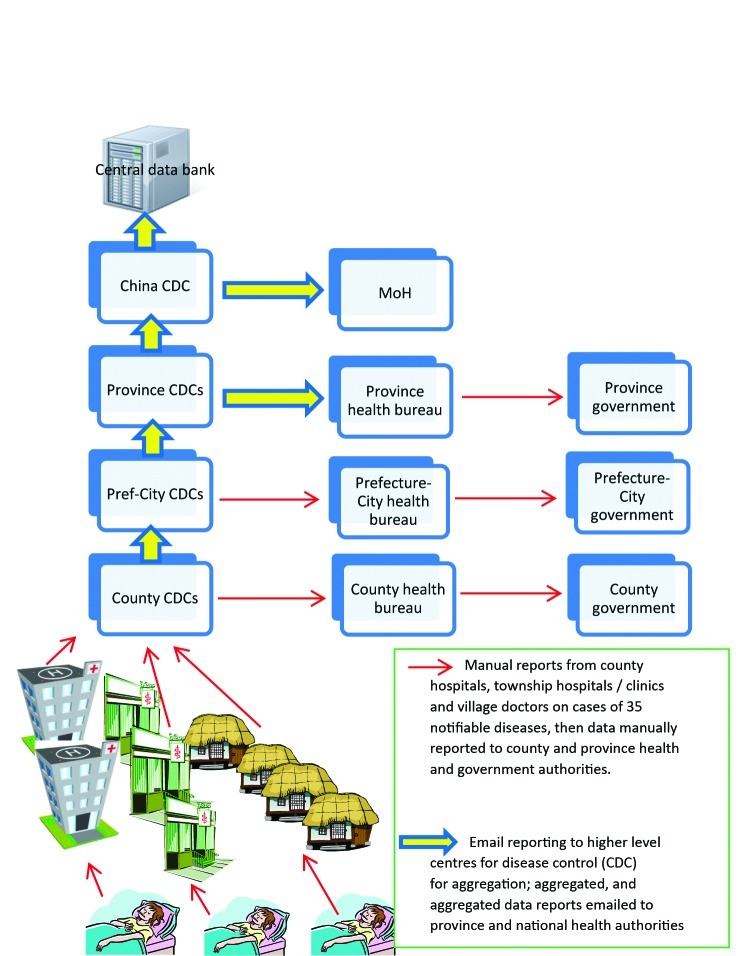
Notifiable disease reporting in China 1985–2003.

In this context, the Ministry of Health had a limited role in initiating and sustaining public health programs. The 1989 Law on Control of Infectious Diseases and associated regulations conferred authority and responsibility to act on local governments, the EPSs, specialized institutes and hospitals (7), but these were weakly implemented. Despite encouraging descriptions of a computerized national disease reporting system and surveillance points and associated auditing (35,36) (**Figure 3**), and the piloting of a model CDC centre in Shanghai from 1998 (37), China did not commence modernization of its public health services until 2002 (38), when the old, mainly academic Chinese Academy of Preventive Medicine and county and provincial EPS network was replaced by a revitalized network of Centres for Disease Control modelled on those in the United States and dedicated to public health. There was no compulsory notifiable disease reporting system until 2004. **Figure 3** depicts the disease reporting system that applied from 1985 to 2003, the years of marketisation and the collapse of coordinated CDC. The system was characterized by poor enforcement and weak oversight; annual reports showed that some health providers and hospitals did not bother to report data. During the early weeks of the Severe Acute Respiratory Syndrome (SARS) in 2003, the multiple treating facilities were either not reporting cases, or were reporting to multiple different and non-coordinated authorities (39).

## Vertical disease control programs replace community approaches to CDC in the 1990s

As indicated, enormous progress was made on CDC in China in the first 30 years of the PRC, so even ignoring the economic reforms it is perhaps not surprising that the approach to CDC changed dramatically after 1980. In the new environment, abstract problems such as those with hygiene and sanitation that caused common, usually non-fatal diseases like diarrhoea and hepatitis now attracted less attention. Indeed, hygiene and sanitation are good examples of public goods whose priority lagged during this period, and China’s progress on the safe water and sanitation indicators for the seventh Millennium Development Goal has been relatively slow (40).

In this new context, the former campaign approach to CDC was replaced by longer term vertical programs, and several related successes in China are documented even during this period when CDC in China was generally marketised. These include elimination of lymphatic filariasis using diethylcarbamazine-citrate fortified cooking salt (41), marked reductions in malaria and control of poliomyelitis (local transmission of which in China was eliminated from 1996 until 2011) and other vaccine-preventable diseases (7,42,43). For the most part, these successes resulted from disease-specific programs, such as the Expanded Program on Immunisation (EPI) and various other long-term projects. A description of two of these priority disease control programs in the context of CDC in China follows.

### Various approaches to the control of schistosomiasis

Schistosomiasis control has been prioritized in China since the 1950s, with various strategies involving coordination between public health, pharmaceutics, agriculture, hydrology, geospatial mapping and animal husbandry experts. The success of this coordination indicates the level of associated political support, but as explained above, this was not always a given. Researchers have also highlighted the impact of farming practices, population movement and China’s economic progress on control of this disease (44). In the 1950s hundreds of millions in 12 southern provinces were at risk of this disease, and around 2% of China’s population was infected (45,46). Early control efforts focused on transmission control, especially by early mass mobilization of people to alter snail habitats (45). With the introduction of praziquantel in the early 1980s (47) the focus changed to morbidity control, and mass treatment funded by a World Bank loan and other activities from 1992-2000 (45). In each case the observed reduction in infection numbers was at risk when priority and funding for control programs declined (46). After completion of the World Bank project, case numbers rose again in certain areas (48); the concentration of cases in poor rural areas and the lack of funding for preventive health care in general led to diminished control efforts, leading national health authorities to rate schistosomiasis control, tuberculosis, hepatitis B and HIV as equally critical priorities, in contrast to its status as a neglected tropical disease in other nations (49). Schistosomiasis persists in seven provinces, in a much smaller area of the upper and lower Yangtze River catchment and particularly in villages whose population totals around three million people (41). National funding was required to kick-start new control efforts including periodic mass chemotherapy, reduction of infection sources (animal management, mechanization of farming, water supply and sanitation measures) and public education, supported by a 2004-2015 government-funded vertical project (49-51). Based on infection rates among the population and cattle in the affected areas, this is apparently the most successful combination of activities yet, and the screening program being undertaken has also demonstrated an impact on rates of infection with the soil-transmitted helminths *Ascaris lumbricoides* and *Trichuris trichiuria*, probably as a result of the sanitation and public education components (51).

### Tuberculosis – persistently high case numbers despite effective diagnosis and treatment

TB is probably the most important communicable disease that China has struggled to control. China has the world’s second highest number of cases of TB (after India) and accounts for 16% of the world’s disease burden. It is estimated that around 45% of the population are infected with *Mycobacterium tuberculosis*, with rates of infection and active disease much higher in rural and western areas; cases number around 1.5 million per year, and deaths around 160 000. Again, TB has been the focus of several large externally-funded projects in China over the last two decades, focusing especially on the introduction and expansion of the five-component Directly Observed Treatment (Short-Course) or DOTS strategy promoted by the World Health Organisation. These were effective in treating patients identified and appropriately referred to dedicated TB facilities, but relatively ineffective in improving case-detection and suffered from many of the same problems as the immunization and schistosomiasis programs. Several reports concluded that there were socio-economic barriers to care-seeking, failure or delay in referring patients for available free treatment (due to loss of income by referring clinicians), weak coordination between hospitals and public health authorities and weak local political and financial prioritization of TB case detection and management (that is, weak co-funding), particularly in poorer counties (52,53). The nature of TB as a disease affecting the poor, the itinerant and those least able to pay for treatment applies in China as elsewhere; absolute case numbers have increased with the population and the problem of multi-drug resistance, currently around 8% of cases, is rising.

Overall TB control in China was another example where CDC suffered due to lack of public funding in poor areas, marketisation of the health sector resulting in lack of patient access to free care, and its handling as a vertical rather than integrated clinical-and-public-health program. More recently, in the context of an overall improvement in CDC in China since 2003, massively increased national funding and improved surveillance for disease using the internet have enabled China to meet and maintain global TB control targets of detecting at least 70% of new sputum-smear positive cases and curing 85% of them (32,54,55). As with schistosomiasis, the increase in national funding for TB control is very encouraging. However, the same challenges continue to apply to TB control as to CDC and public health in China in general: national and local funding for dedicated and trained staff and services, and making related services accessible and affordable to all, including the mobile population, despite the continued focus on profit in most health facilities.

### Control of sexually-transmitted diseases – China’s newest vertical CDC program

By contrast to the targets of vertical disease-control initiatives, sexually transmitted diseases (STDs) have re-emerged as a major priority in China due to the lack of such a program. China’s legendary success in controlling STDs during the 1950s and 1960s was due to a combination of socialization (in which STDs were portrayed not typically as a sign of “bad behaviour” but as a legacy of the old bourgeois and exploitative society, particularly with respect to women); treatment (destigmatising syphilis and gonorrhoea made mass screening and drug treatment relatively easy), and socio-economic approaches (the banning of prostitution, emancipation of women and creation of employment for poor women) (4,6,10,11). This combination was inseparable from the revolutionary milieu of the time, and despite very high rates of infection during the early years of the People’s Republic, helped to “eliminate” STDs from China by 1964 (10,56). This situation prevailed until the liberalization of commerce, movement, social customs and secular changes in sexual behaviour allowed the reappearance of STDs in the 1980s (39,56). There were massive increases in STD incidence and an emerging HIV problem in China in the 1990s (57), and the same problems that have led to difficulties in sustaining control of TB and schistosomiasis have plagued STD control: lack of knowledge of disease prevention and treatment, including HIV, among the poor and some echelons of the health sector (58); lack of physical and financial access to good care, along with profiteering by health providers; lack of funding for screening programs, and poor coordination across sectors (including within the health sector, between MCH staff and other clinicians), creating an urgent problem (59,60). According to a former director at China’s National Centre for Women’s and Children’s Health, in 2008: “In the past fifteen years, the prevalence of congenital syphilis increased by 2000 times in China, excluding foetal deaths, still-births and abortions caused by syphilis during pregnancy. Surveillance data reveal the incidence of congenital syphilis increased at the rate of 72% each year from 0.01 in 1991 to 35 in 2006 per 100 000 live births” (Wang Linhong, former Director, National Centre for Women’s and Children’s Health at China CDC, personal communication). Syphilis is now numerically the third most common reportable infectious disease in the PRC (behind viral hepatitis and TB) (Dr Yang Weizhong, China CDC, personal communication). To its credit, the government has again massively increased funding for education, screening and treatment of STDs, including HIV (60), but the long term success of these measures will again depend on the level of uptake of these activities, fair access to care and local government support.

## China’s wake-up call on CDC: improvements since SARS and health system reform

For both TB and schistosomiasis, it is evident that cessation of internally- and externally-supported disease control programs in the early 2000s was a major setback. Outside the academic and public health community in China, interest to fund and implement programs to control specific diseases associated with poverty and under-development was low at this time. As a result, despite improvements in nutrition, socio-economic status and health infrastructure, there was little progress in infectious disease rates and suggestions that some were increasing slightly during this period (30), although it is likely that this also reflected improved surveillance and diagnosis (39). What was undoubted, however, was the increasing urgency of major reform to CDC and China’s health sector in general (29,61-63) due to worsening equity (21, 64-66), a high level of public complaint and government acknowledgement of the problem. Crystallising the situation in the most humbling way came the SARS outbreak in early 2003, which forced China’s government and health authorities to act quickly and decisively on the dangerous situation with respect to CDC and, albeit more slowly, on the reform of the health sector.

**Figure 4:**
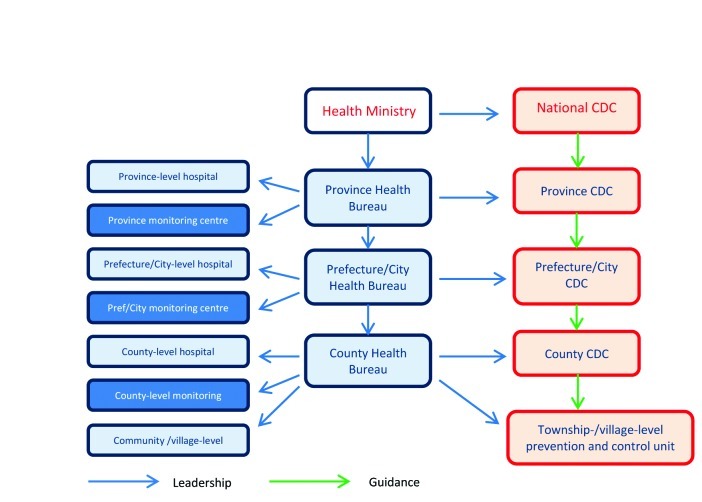
China’s new infectious disease prevention and control system, introduced in 2004 (after Yang Weizhong, personal communication, 2010).

Much has been written about China’s initial denial of the extent of the SARS outbreak (67), and the implications for its control (68). The events occurred despite preceding attempts to renovate the EPSs, as described above, but there is no denying that China remained grossly ill-equipped to deal with a disease of this nature in 2003, and government hugely increased its support for CDC (physical infrastructure, staffing and funding) after this shock (39). Two other major CDC-related impacts of SARS in China were undertaken. First was the revision of the Law on Infectious Diseases in August 2004, mandating the reporting of 37 notifiable conditions, including immediate reporting of certain diagnoses and replacing a system which had essentially become optional and mainly answerable to local government, not the CDC hierarchy. As a result, in restoring its population health objectives CDC was mainstreamed in China’s health sector, with both the curative and disease-control sectors responsible for prevention, reporting and management of infectious diseases (Dr Yang Weizhong, China CDC, personal communication) (**Figure 4**).

**Figure 5:**
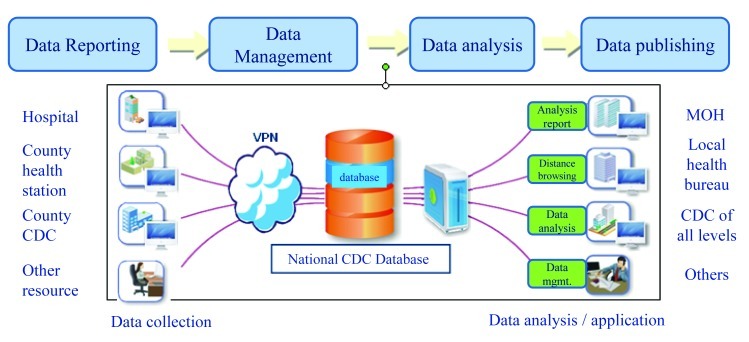
China’s web-based notifiable infectious disease direct reporting system (Yang Weizhong, China CDC, permission to reproduce received).

Second was the development of a new electronic notifiable disease reporting system to answer the central government’s request for a case-based, national, integrated and web-based system (incorporating notifiable diseases, risk factors, emergencies and also specific systems for reporting certain diseases like TB, influenza, plague and HIV). In contrast to the old system of weekly and monthly consolidated reports, the new system uses the internet rather than email to upload disease information, not only from local CDC facilities but also from hospitals and health inspection agencies, enabling analysis of data pertaining to reportable diseases and identification of disease outbreaks and trends in real time (**Figure 5**).

**Figure 6:**
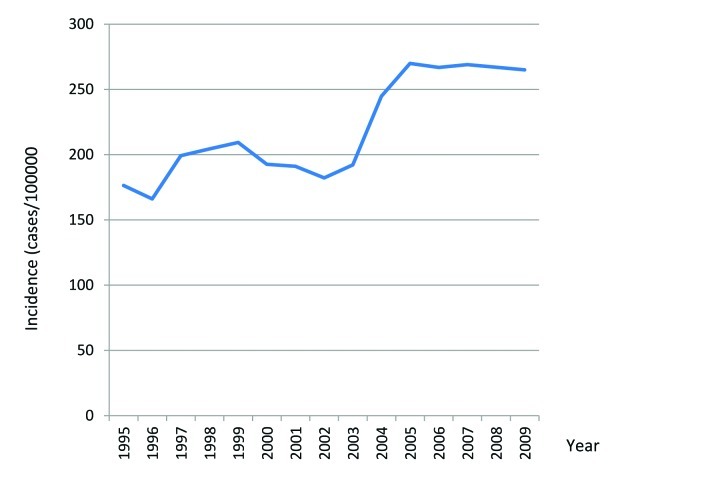
Improved notifiable disease reporting since introduction of real-time system in 2004 (after Wang (39) and data presented at an International Symposium on Research and Control of Infectious Diseases of Poverty, Shanghai, China, 2010).

Again, the mandating of hospital reporting drew the clinical sector into CDC as never before, raising clinicians’ awareness on the public health significance of their actions on infectious diseases and population health; the coverage of this reporting system in 2009 was 100% of CDC-facilities, 97.8% of county-level or higher hospitals and 83.8% of township/village-level facilities, up from 66% in 2007, and the delay in reporting of and entering a notifiable disease report is reported to have dropped from almost 5 and 3.5 days, respectively, to less than one day (Dr Yang Weizhong, China CDC, personal communication). Additional surveillance continues through the notifiable disease reporting system and specific surveillance systems for HIV/AIDS and other STDs, TB, EPI target diseases (for example, for acute flaccid paralysis and measles) and others. The impact of these two initiatives is evident in the rise in the number of notifiable disease reports since 2003 (39) (**Figure 6**).

Alongside these two broad CDC initiatives, a number of disease-specific, donor- and particularly government-funded initiatives have also demonstrated an increased commitment to CDC in China. These include massive increases in funding for control of TB, schistosomiasis, malaria and STDs; treatment and prevention of maternal-to-child-transmission of HIV/AIDS; prevention, screening and treatment of other STDs; vaccine-preventable diseases (such as control of measles through various provincial campaigns and a national campaign in September 2010; control of hepatitis B through catch-up vaccination of older children; expansion of routine immunization to cover 12 antigens since 2007; an enormous program of subsidies to encourage hospital delivery and prevention of neonatal tetanus (also enabling dramatic increases in birth-dosing with hepatitis B vaccine) and introduction of a national child immunisation registration and information system); infectious disease surveillance during emergencies (including use of mobile phones to report on disease incidence in the areas affected by the Sichuan earthquake) and public education campaigns and research to reduce the risk of emerging threats such as recrudescence of dengue fever; increases in brucellosis, zoonoses and the impact of annual outbreaks of influenza and EV71 infection (data available upon request). Both GAVI and the Global Fund for AIDS, TB and Malaria have also supported large scale CDC activities in China in recent years.

These developments have since 2009 been taking place in the context of other major developments in China’s health sector, some of which are likely to directly benefit CDC. Among the initiatives being rolled out as part of China’s health system reform (HSR) are a 15 (now 25) yuan-per-capita public health subsidy for grassroots-level providers, to facilitate their implementation of nine public health activities at village level; including health promotion and implementation of CDC; a National Essential Drugs Scheme intended to control prescribing practices and profiteering by village and township doctors, including in the treatment of infectious diseases (26), and even more funding to improve the staffing and physical infrastructure of China’s health system (69).

## Risks and challenges

There is no doubt that China is in a much better position to handle another disease outbreak like SARS; indeed, the response to the ongoing highly-pathogenic H5N1 and 2009 H1N1 influenza outbreaks, despite accusations of under-reporting and heavy-handed quarantine of travellers, demonstrate China’s increased capacity and intention to act quickly, decisively and in unison across national, provincial and county levels on CDC when population health is threatened.

In fact, the major reasons for slow progress in some aspects of CDC overall is not unique to CDC, nor to China. Decentralisation of the funding and implementation of many health programs in China and elsewhere, although forced upon governments by economic reality and the need to build capacity and encourage the taking of responsibility, is inimical to consistent, reliable and robust outcomes. To the extent that China is relying on poor, predominantly rural provinces and counties for CDC, the wait for elimination of infectious diseases dependent on more than drugs and vaccines may be a long one.

**Figure 7:**
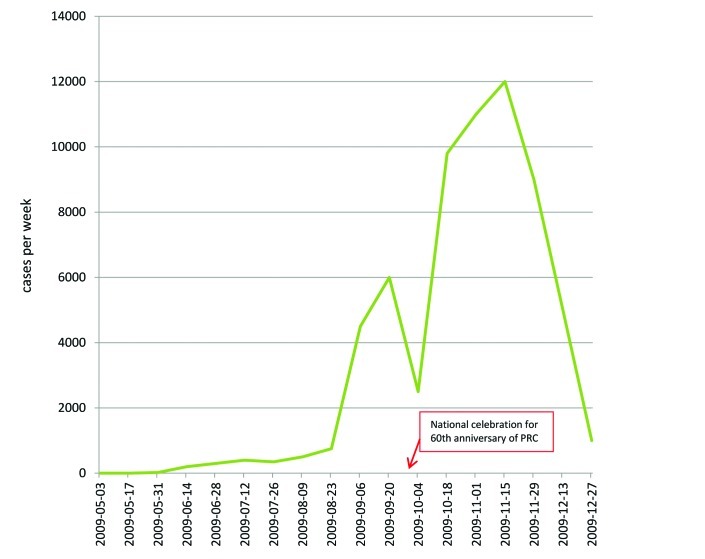
H1N1 case numbers in China by fortnight, May – December 2009 (based on data presented at an International Symposium on Research and Control of Infectious Diseases of Poverty, Shanghai, China, 2010). PRC – People’s Republic of China.

Another problem, also not unique to China but perhaps less tolerable in a nation of its size and importance to global health, is the opacity of the situation at certain times. Despite marked improvements in disease surveillance and CDC since SARS, a remarkably similar and concerning reluctance to report disease outbreaks in times of political sensitivity persists. Recent examples include the likely cover-up of the melamine scandal before the Olympic Games in 2008 (70), and the probable under-reporting of cases of H1N1 influenza just prior to the celebration of the 60th anniversary of the People’s Republic in October 2009 (**Figure 7**). The ongoing tendency of those in power in China to put nationalism and politics ahead of public health at certain key times suggests a continuing risk for CDC (71).

China has not yet taken up global recommendations to vaccinate all children against rotavirus, pneumococci, *Haemophilus influenza* type b and human papillomavirus. Although the national incidence of rotavirus diarrhoea is almost certainly lower than previously thought (72), a case could easily be made for introduction of the vaccine in poorer provinces or in rural areas, on mortality, morbidity and possibly economic grounds. The same could be said for the two respiratory pathogens, but the data are scant and there has been a long-standing reluctance to introduce these vaccines in China, for two reasons: first, given that China does not use any of the newer combination vaccines it will further complicate an already-crowded vaccination schedule; second and more important, local manufacture of most of these vaccines has not yet commenced, and China does not use imported vaccines in its EPI. These and other so-called category B vaccines are available for private purchase from CDC facilities across China, but there are no data on coverage. It is safe to assume that those who would benefit from them most do not receive them.

Other risks for CDC in China have recently been studied by experts and some are perceived to remain significant. These include the risk of population mobility, persistent proximity of humans and animals in some areas, the regular appearance of new strains of influenza and other pathogens in China, behaviour changes impacting on STDs and the continued low standard of clinical care in poor areas (68,73).

Finally, TB is not the only bacterium for which antibiotic resistance is a major emerging problem in China. Marketisation of the health sector, all the way down to village level, resulted in massive overuse of antibiotics, and a very active pharmaceutical manufacturing sector has avidly promoted “new, improved” drugs to health providers across the nation. Although data are hard to come by as clinical microbiology is a luxury not usually purchased by health services in China, it is safe to assume that multi-resistant bacteria are common in China, and pose a threat to CDC in clinical settings.

## Conclusion

The study of CDC in China provides a fascinating opportunity to understand the early tribulations and achievements of the People’s Republic, during which time the top-down campaign-style approaches adopted from the Soviet Union were replaced by a bottom-up approach led by village doctors, supported by township and county cadres and funded by the CMS. The introduction of a market economy, with the breakdown of these grassroots structures and the reliance on vertical programs has challenged CDC in China. Changes to reporting and the structure and priority of CDC after SARS, along with more recent reforms of the health sector and injection of new funds for disease control programs, allows reasonable expectations of further progress in CDC in the world’s largest nation.
